# Quantification of Mesenchymal Stem Cell Growth Rates through Secretory and Excretory Biomolecules in Conditioned Media via Fresnel Reflection

**DOI:** 10.3390/s131013276

**Published:** 2013-09-30

**Authors:** Harith Ahmad, Kavintheran Thambiratnam, Ahmad Z. Zulkifli, Anthony Lawrence, Ali A. Jasim, Wijenthiran Kunasekaran, Sabri Musa, Nareshwaran Gnanasegaran, Punitha Vasanthan, Pukana Jayaraman, Noor H. A. Kasim, Vijayendran Govindasamy, Mohammad S. Shahrir, Sulaiman W. Harun

**Affiliations:** 1 Photonics Research Centre, University of Malaya, 50603 Kuala Lumpur, Malaysia; E-Mails: harith@um.edu.my (H.A.); zain_73@yahoo.com (A.Z.Z.); a_a_jasim@yahoo.com (A.A.J.); swharun@um.edu.my (S.W.H.); 2 Regenerative Dentistry Research Group (ReDReG), University of Malaya, 50603 Kuala Lumpur, Malaysia; E-Mails: dr.anthony.lawrence@gmail.com (A.L.); wijenthiran@yahoo.com.sg (W.K.); sabrim@um.edu.my (S.M.); naresh_waran_28@hotmail.com (N.G.); srinitha_37@yahoo.com.my (P.V.); pukana83@yahoo.com (P.J.); nhayaty@um.edu.my (N.H.A.K.); 3 Research and Development Department, Hygieia Innovation Sdn. Bhd, Lot 1G-2G, Komplex Lanai, No.2, Persiaran Seri Perdana, Presint 10, 62250 Federal Territory of Putrajaya, Malaysia; E-Mail: vijay@hygieiagroup.com; 4 Institute of Mathematical Sciences (ISM), Faculty of Science, University of Malaya, 50603 Kuala Lumpur, Malaysia; E-Mail: mshazri@gmail.com

**Keywords:** fresnel sensor, biosensor, mesenchymal stem cells, real-time measurement, Wharton's Jelly, conditioned media, cytokines

## Abstract

An efficient and low cost optical method for directly measuring the concentration of homogenous biological solutes is proposed and demonstrated. The proposed system operates by Fresnel reflection, with a flat-cleaved single-mode fiber serving as the sensor probe. A laser provides a 12.9 dBm sensor signal at 1,550 nm, while a computer-controlled optical power meter measures the power of the signal returned by the probe. Three different mesenchymal stem cell (MSC) lines were obtained, sub-cultured and trypsinized daily over 9 days. Counts were measured using a haemocytometer and the conditioned media (CM) was collected daily and stored at −80 °C. MSCs release excretory biomolecules proportional to their growth rate into the CM, which changes the refractive index of the latter. The sensor is capable of detecting changes in the number of stem cells via correlation to the change in the refractive index of the CM, with the measured power loss decreasing approximately 0.4 dB in the CM sample per average 1,000 cells in the MSC subculture. The proposed system is highly cost-effective, simple to deploy, operate, and maintain, is non-destructive, and allows reliable real-time measurement of various stem cell proliferation parameters.

## 1. Introduction

Optical fiber based sensors have long been the focus of substantial research to develop a light-weight, cost effective and highly accurate sensor for measuring various parameters such as temperature, strain and pressure [1–6]. Recently however, optical fiber sensors are beginning to find use for a variety of new and unique applications, one of the most interesting of which is the detection and measurement of various parameters from biological constituents, such as the presence of particular constituents, constituent concentrations and growth rates [7,8]. Fiber-based sensors are particularly advantageous for this task, as they are lightweight and compact, and can also be easily multiplexed, thus reducing the cost and complexity of the system. Furthermore, they do not require a medium for sensing, thereby allowing them to operate in sensitive environments [9–10].

A key application of fiber based sensors would be in the measurement of cell growth in real time. Most current approaches for measuring cell growth such as haemocytometers, although highly accurate, are unfortunately also destructive in nature. As such, multiple cell cultures must be seeded for each count, increasing the cost and complexity of the experiments. At the same time, these approaches do not allow for measurements to be carried out in real-time, thereby negating potentially important experimental information. One current approach for measuring stem cell counts is by using digital holographic microscopy. This experimental system however consists of many optical components, which use elaborate methods such as coherent monochromatic sources and microscope objectives to collect the object wavefronts as well as complex image processing techniques [11,12]. Alternative methods under research include surface plasmon resonance [13] and polarization birefringence [14]. In addition to this, other avenues of research are investigating the potential of using planar optical waveguides based on the evanescent optical field that allow the number density to be measured [15] or the use of modulated coupled light in a waveguide that allow for the investigation of the human embryonal carcinoma stem cells [16].

In this work, an optical fiber sensor, operating on the principles of Fresnel reflection, is proposed and demonstrated as a means to measure the growth rates and cell quantities from three stem cell lines. The proposed sensor has many advantages over traditional measurement techniques, as it does not entail the destruction of the cells during the counting process, allowing them to be used for further experimentation. Furthermore, the sensor is only minimally invasive, and can thus be used to provide real-time measurements of the sample of interest. This is the first time, to the best knowledge of the authors, that a Fresnel based fiber sensor has been used in the determination of cell numbers and growth rates in real time.

## 2. Stem Cell Morphologies

### 2.1. Determination of Stem Cells

Although embryonic stem (ES) cells have, and for that matter are still widely regarded as having better therapeutic potential than any other stem cell line, the significant constraints associated with ES cells, arising from legal restrictions and ethical ramifications severely limits their use in translational medicine [17]. As a result of this, mesenchymal stem cells (MSCs) have now become the preferred source of stem cells for research purposes. MSCs have long-term *ex-vivo* proliferation, multi-lineage potential [18] and also immunomodulatory properties [19], making them a promising candidate for replacing ES cells in regenerative stem cell therapy. For this work, two stem cell lines, namely Wharton's Jelly stem cells (WJSCs) and deciduous dental pulp stem cells (DPSCs) were chosen to be cultured, taking into consideration factors such as availability as well as its potential in regenerative stem cell therapy.

Wharton's Jelly is the embryonic mucous connective tissue lying between the amniotic epithelium and the umbilical vessels, and WJSC samples are obtained from the umbilical cord, which is discarded together with the placenta after birth. As such, WJSC cells can be construed as an inexhaustible and non-controversial type of stem cell. WJSCs bear a higher proliferation/differentiation rate and regenerative ability compared to adult tissue MSCs. The potential emerging clinical applications of WJSCs have been shown in multiple specialties, such as in treating graft versus host disease (GVHD) and systemic lupus erythematosus (SLE) [20], as well as assisting in cardiac function following myocardial infarction in rat models [21]. DPSCs were identified as new post-natal stem cells, which have the ability to regenerate into adipocyte and neural-like cells [22]. Stem cells from dental sources were first isolated from human pulp tissue more than a decade ago [23], which subsequently spurred a concentrated effort into analyzing the therapeutic properties of periodontal ligament stem cells (PDLSCs) [24], stem cells from apical papilla (SCAP) [25] and DPSCs [26].

The characterization of mesenchymal stem cells is based on the “gold standard” criterion, which was established for bone marrow mesenchymal stem cells (BMMSCs) [27]. MSCs from dental origins could be differentiated into either pancreatic or neural cell lineages, besides fulfilling the three lineage differentiation criteria: adipogenic, chondrogenic and osteogenic [26]. In comparison to BMMSCs, dental-derived stem cells are more accessible and non-invasive. Extensive research on the pluripotent stability of ES cells and *in vivo* understanding the biology of these dental stem cell populations is a prerequisite towards knowing the extent of their efficacy for future therapeutic usage. It is here that the problem lies—typical MSC measurement techniques require that the cell cultures be trypsinized and re-suspended in trypan blue dye. The tryphan blue only stains living cells, so that it can be easily detected by systems such as a haemocytometer, but once the cells have been stained they cannot be used any further and are discarded. The remaining unstained trypsinized cells are then re-cultured in subsequent subcultures, and while this may serve to ensure the continued proliferation of cells form the primary culture, it has to be noted that mesenchymal stem cells (MSCs) in later subcultures will lose their differentiation potential and pluripotency [28], thus affecting the outcome of the experiment.

### 2.2. Preparation of Samples

Sound intact deciduous stem cells (SCDs) were extracted from individual patients (aged 6–11, n = 3) who were undergoing planned serial extraction at the Department of Children's Dentistry and Orthodontics, Faculty of Dentistry, University of Malaya. The SCDs were obtained using procedures as previously described [28]. Human umbilical cords were collected from full-term births after either cesarean section or normal vaginal delivery (age = 28–35, n = 3) with informed written consent as per the approved guidelines by the Malaysian Ministry of Health. The WJSC samples were extracted/obtained from the umbilical cords as previously described [29] and stored under a protocol that has been approved by the Medical Ethics Committee, Faculty of Dentistry, University of Malaya (Medical Ethics Approval Number DFCD0907/0042(L)). The study was conducted with these samples, and all experiments were repeated independently to ensure reproducibility of results.

### 2.3. Isolation & Cell Culturing

Dental root surfaces were sterilized externally with povidone iodine and the pulps were extirpated within 2 h after extraction and processed. The pulp tissue was minced into small fragments before digestion in a solution of 3 mg/mL collagenase type I (Gibco, Grand Island, NY, USA) for 40 min at 37 °C. Similarly, the WJSC tissue samples were minced into small fragments before digestion in a 3 mg/mL solution of collagenase type I for 40 min at 37 °C. All samples were then neutralized with 10% of fetal bovine serum (FBS) (Hyclone; ThermoFisher Scientific Inc, Waltham, MA, USA), centrifuged and were seeded in T25 culture flasks with conditioned media containing DMEM-KO Basal media (Invitrogen, Carlsbad, CA, USA), 0.5% 10,000 mg/mL penicillin/streptomycin (Invitrogen); 1% Glutamax (Invitrogen) and 10% FBS, with humidified atmosphere of 95% of air and 5% of CO_2_ at 37 °C.

The cells were typsinized prior to 80% confluence and processed for subsequent subcultures (SC1, SC2, SC3, SC4 *etc.*). Three sub-cultures, Deciduous Dental Stem Cell Subculture 3 (SCD.SC3), Deciduous Dental Stem Cell Subculture 4 (SCD.SC4) and Wharton's Jelly Stem Cell Subculture 3 (WJSC.SC3) were chosen for experimentation and were plated at a seeding density of 1,000 cells/cm^2^. The cultures were allowed to grow in standard growth conditions during the experiment duration of 9 days, and each day viable cell counts were performed using a haemocytometer and 0.4% trypan blue dye, with each sample undergoing repeat counts to reduce error. The cell counts obtained by the haemocytometer is used to verify readings obtained by the proposed sensor. Simultaneously, on Day 1 through Day 9, conditioned media (CM1 through to CM9) was collected from each subculture in triplicate fashion (Day 1 = {R1, R2, R3}, Day 2 = {R4, R5, R6}, Day 3 = {R7, R8, R9}, *etc.*) placed into vials before being stored at −80 °C, resulting in a total of 27 samples for each subculture and an overall total 81 samples for testing. The average cell count obtained, together with the standard deviation (SD) for each count, is given in Table 1.

**Figure 1.**
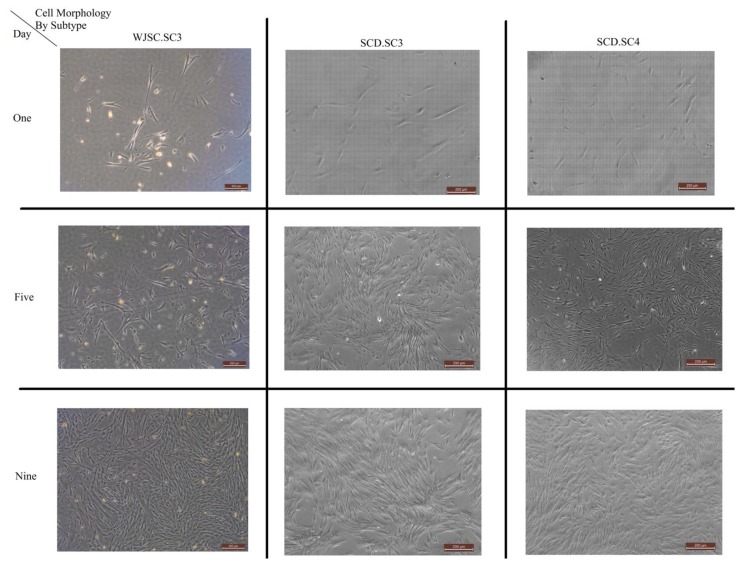
Morphology and the growth of the different MSCs obtained at different days of the experiment. The captured images provide a visual validation of the cell counts obtained in Table 1.

Figure 1 shows the morphology growth of the sub-cultures, divided by type of sub-culture and taken at Days 1, 5 and 9. The images are captured using a Leica DM16000B inverted phase contrast microscope.

A standard conditioned media (designated as CM0) which included DMEM-KO, 10% FBS, 1% Glutamax™, and 0.5% 10,000 mg/mL penicillin/streptomycin was used as the control for experimentation.

## 3. Experimental Setup

**Figure 2.**
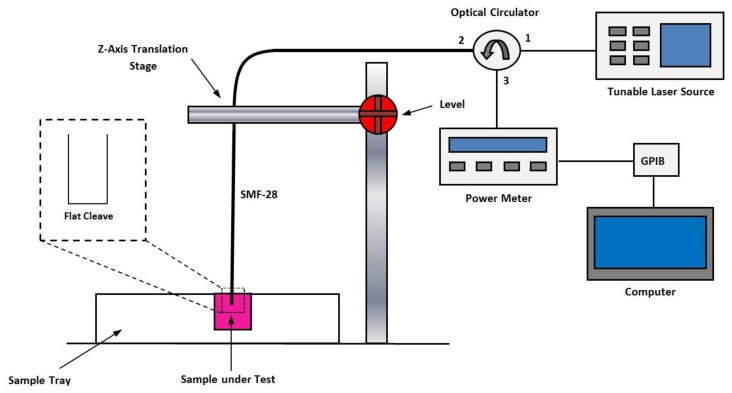
Experimental setup of the proposed sensor system.

The setup of the proposed optical sensor system is given in Figure 2. A Yokogawa AQ2200-136 tunable laser source (TLS) acted as the signal source for the sensor, and was connected to Port 1 of an optical circulator (OC). Port 2 of the OC was connected to a 1 m long single-mode fiber (SMF-28) with a flat cleaved end. The flat cleaved end served as the sensor by means of Fresnel reflection [30] while the remaining length of the SMF-28 served as a bi-directional transmission medium for the sensor signal. A Nivoc A1362M Universal Clamp and ND121 Clamp Holder translation stage, capable of moving in the vertical axis, was used to lower and raise the sensor head into the sample being tested. The translation stage allowed for the complete immersion of the sensor head. The immersion depth was deemed immaterial so long as the face of the fiber was completely immersed in the conditioned media samples; however, to maintain consistency the translation stage was used to ensure that the immersion depth was 2 mm. Port 3 of the OC was connected to an ILX Lightwave OMM-6810B optical power meter (OPM). The OPM was equipped with a general-purpose interface bus (GPIB) interface, and was connected to a LabVIEW equipped computer to automate the data acquisition process.

The TLS was used to generate a 1,550 nm sensor signal at a power of 12.9 dBm, which traveled to Port 1 of the OC through Port 2 towards the sensor head. At the sensor head, the difference in the refractive index ratio between the glass and liquid interface resulted in a portion of the sensor signal being reflected back into the SMF-28. The reflected signals then traveled back along the SMF-28 fiber where it re-entered the OC via Port 2 and was emitted at Port 3, which was connected to the OPM and computer. The computer was programmed to obtain power readings from the sensor at one-second intervals for a period of 10 s for each sample. The sensor was first calibrated using unused conditioned media, where a reflected power of 11.5 was obtained, corresponding to a loss of 1.4 dBm, or a −log_10_ (loss) of −0.14 dBm. This value was used to calibrate the zero value of the sensor, and all subsequent results were taken using this value as the base-line.

Following this, the triplicate conditioned media samples from each stem cell lines were measured on a daily basis. The power loss was measured and all obtained data was then compiled for further analysis.

## 4. Results and Discussion

**Figure 3.**
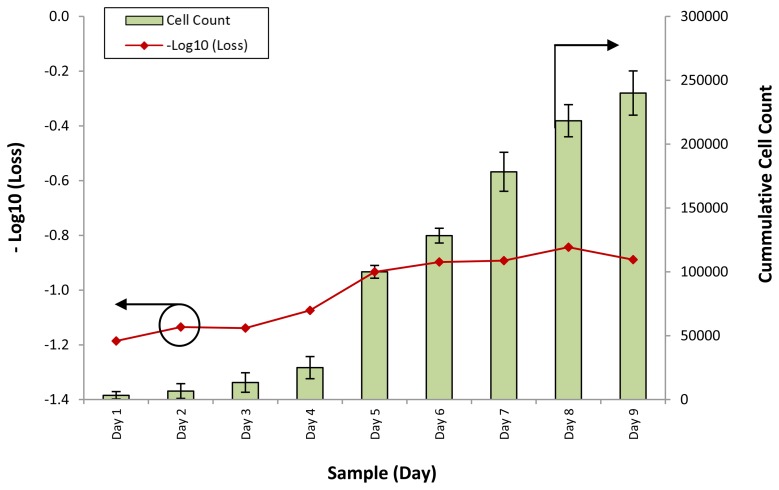
−Log_10_ (Power Loss) and cumulative cell count against different conditioned media samples for the WJSC.SC3 cell line. The power loss is represented by the line graph, with the corresponding X-axis on the left, while the cell count is represented by the bar chart, with the corresponding X-axis on the right side of the graph.

The response of the sensor towards the different samples for all cell lines was computed in terms of the loss of power for each different sample. Figure 3 shows the loss of optical power against different samples of the WJSC.SC3 cell line. The cell counts for the corresponding samples are also provided.

It can be seen that the response of the sensor, in the form of −Log_10_ (Loss), is similar to the growth rate of the cumulative cell count. At the initial stage of the experiment, from days 1 to 4, the response of the sensor rises slightly, from a value of −1.2 to about −1.1 as the cumulative cell count increases from 3,333 to 2,500. However, on Day 5, the cumulative cell count increases substantially to approximately 100,000, with a corresponding increase in the −Log_10_ (Loss) readings of the sensor to −0.9. Further increases in the cumulative cell count also result in changes in the response of the sensor, which increases to between −0.8 and −0.9 on the last days of the experiment.

The power loss of the sensor is a function of the refractive index of the conditioned media, which itself is a function of the density and composition of the conditioned media. The conditioned media serves both as a source of nutrients as well as a suspension medium upon which the cells can be cultured, thus as the cells proliferate, the nutrient content of the conditioned media is depleted. Concurrently, excretory and secretory substances are released by the mesenchymal stem cells into the conditioned media, changing its overall composition. This in turn affects the refractive index of the conditioned media. Based on this model, and with the results of Figure 3, it can be seen that the average power loss from the conditioned media is generally inverse to the cell count, with the power loss decreasing by 0.4 dB in the conditioned media for an average increase of 1,000 mesenchymal stem cells. The power returning from the face of the fiber can be given as:

$$R={\left(\frac{n_{1}-n_{o}}{n_{1}+n_{o}}\right)}^{2}$$

where:

**R** is the fraction of incident light reflected by the face of the fiber,
**n_1_** is the refractive index of the fiber core (1.45 for most commercially available fibers) and
**n_o_** is the refractive index of the conditioned media.

**Figure 4.**
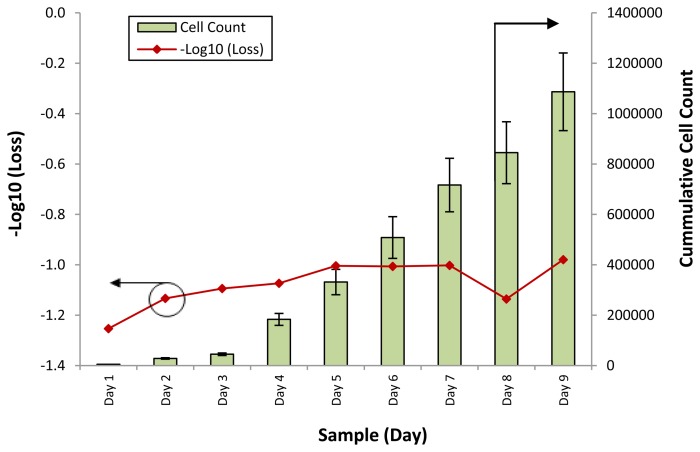
−Log_10_ (Power Loss) (dB) and cumulative cell count against different conditioned media samples for the SCD.SC4 cell line. The power loss is represented by the line graph, with the corresponding X-axis on the left, while the cell count is represented by the bar chart, with the corresponding X-axis on the right side of the graph.

**Figure 5.**
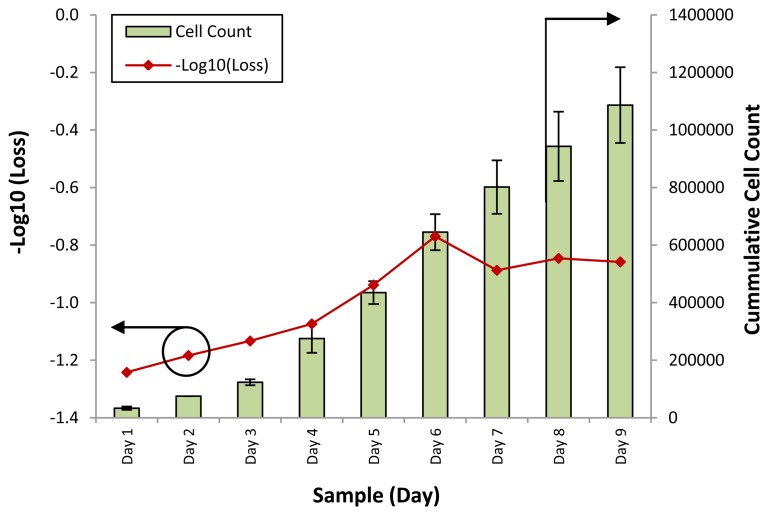
−Log_10_ (Power Loss) (dB) and cumulative cell count against different conditioned media samples for the SCD.SC3 cell line. The power loss is represented by the line graph, with the corresponding X-axis on the left, while the cell count is represented by the bar chart, with the corresponding X-axis on the right side of the graph.

Therefore, the loss of power can be seen as being caused by a change in the refractive index of the conditioned media. Figure 4 shows the loss of optical power against different samples of the SCD.SC4 cell line, while Figure 5 shows the loss of optical power obtained by the fiber sensor when tested with samples from the SCD.SC3 line. The cell counts for the corresponding samples are also provided for both graphs. Testing with the SCD.SC4 and SCD.SC3 lines was carried out to gauge the performance of the sensor when used on different cell lines, such as WJSC and SCD.SC4, as well as the performance of the sensor in differentiating cell counts from cell lines derived from similar cell types, such as SCD.SC4 and SCD.SC3. It can be seen in both cases that the response of the fiber sensor is that in most cases is behaves in a similar fashion, with an inverse power loss relationship between the secretory and excretory biomolecules within the conditioned media to the cell count.

For the SCD.SC4 cell line, the initial low cell counts of between 5,000 to 17,000 cells from Day 1 to Day 3 gives a corresponding of −1.3 dB at Day 1 and decreasing to −1.1 dB at Day 3. As the cumulative cell count increases to more than 1 million on the last day of the experiment, the response of the sensor continues to increase steadily, to a value 0f −0.9. The overall response of the third cell line (SCD.SC3) is also similar, with the low cell count on the first three days corresponding to a high signal loss, from 17.4 dB on Day 1 and decreasing to 13.6 dB on Day 3. As with the SCD.SC3 cell line, a spike in the cell count is observed in Day 4, with the cell count rising to more than 150,000 cells and increasing to above 210,000 cells on Day 6. The corresponding loss of power over these three days decreases linearly as well, from 11.5 dB to 5.9 dB. After Day 6, the cell count decreases, to around 156,000 cells on Day 7, and approximately 141,000 and 143,000 cells on Days 8 and 9. The loss of power during this time is relatively constant, rising from 5.9 dB to approximately 7.7 dB on day 7 and remaining so until the last day of measurement.

**Figure 6.**
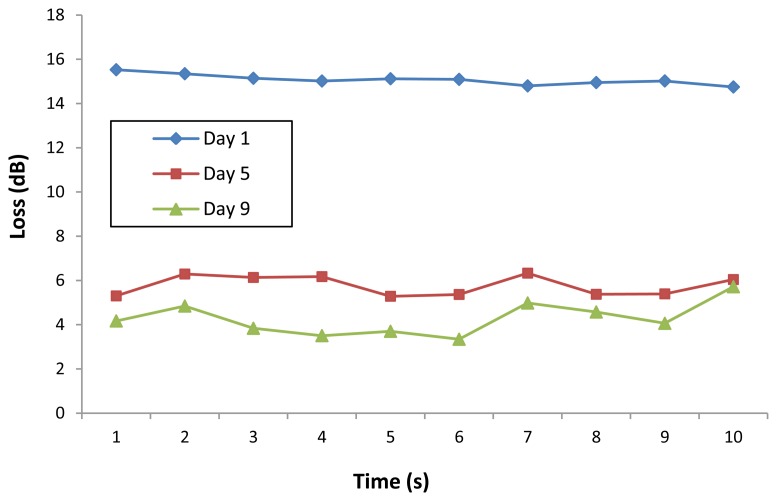
Power loss (dB) against time for selected samples for the WJSC.SC3 cell line (samples taken from Day 1, Day 5 and Day 9).

**Figure 7.**
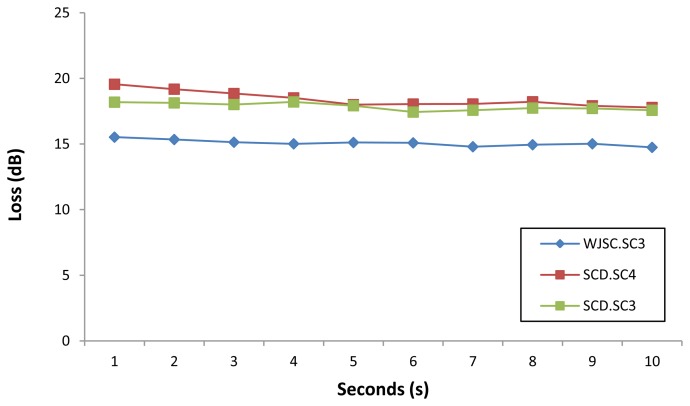
Power loss (dB) against time for selected samples for the three different cell line (all samples taken from Day 1).

Figure 6 shows the loss of power measured over a period of 10 seconds for selected samples of the WJSC.SC3 cell line. It can be seen that the sensor is very stable, with only minor fluctuations in the loss readings obtained from the sensor. A similar performance is also observed for loss measurements taken from selected samples of the three different cell lines, as shown in Figure 7.

Overall, a general pattern can be observed between cell proliferation and loss of power. This is due to production of various secretory and excretory biomolecules by the proliferating cells, including autocrine and/or paracrine factors and also cytokines specific to these cell lines. It is well documented that MSCs produce many paracrine factors that help to maintain their pluripotency [31]. These biomolecules will increase as the number of the cells increase and will contribute to more power loss, as these biomolecules will increase the absorbance rate. We postulate that this loss of power should hold true for all living cells that secrete and excrete biomolecules. We further postulate that each measured cell line/type will produce a different amount of power loss due to the variance in quantity as well as type of biomolecules secreted and excreted during cell proliferation. As such, with further testing of different cell lines and cell types using the experimental setup with manual confirmation using traditional methods (*i.e.*, a haemocytometer), a database can be established wherein the expected power loss corresponds to the current growth rate of any particular cell line. The use of data and single processing techniques, modified from those used in references [32,33] will also be able to improve the performance of the sensor significantly.

This method will have significant advantages over current measurement methods. Firstly, with reference to the baseline-established specific for MSCs in a particular subculture, real-time proliferation (*i.e.*, growth rate) of the culture can be measured. Secondly, abnormalities in the culture due contamination (e.g., due to Mycoplasma sp.) or stress can be identified should the growth rate not correspond to an established/baseline growth rate.

## 5. Conclusions

An efficient and low cost optical method for directly measuring the concentration of homogenous biological solutes is proposed and demonstrated. The proposed system operates by Fresnel reflection, with a flat-cleaved single-mode fiber serving as the sensor probe. A laser provides a 12.9 dBm sensor signal at 1,550 nm, while a computer-controlled optical power meter measures the power of the signal returned by the probe. Three different MSC lines were obtained, subcultured and trypsinized daily over 9 days. Counts were measured using a haemocytometer and the CM was collected daily and stored at −80 °C. MSCs release excretory biomolecules proportional to its growth rate into the CM which changes the refractive index of the CM. The sensor is capable of detecting changes in the number of stem cells via correlation to the change in the refractive index of the CM, with the measured power loss decreasing approximately 0.4 dB in the CM sample per average 1,000 cells in the MSC subculture. The proposed system is highly cost-effective, simple to deploy, operate, and maintain, is non-destructive, and allows reliable real-time measurement of various stem cell proliferation parameters.

## Figures and Tables

**Table 1.** Averaged cell count, by day, for each cell line. The standard deviation for each count is delineated by the SD columns.

**Day**	**WJSC.SC3**	**SD**	**SCD.SC3**	**SD**	**SCD.SC4**	**SD**
1	3,333	2,886.751	33,333	5,773.502	5,000	0.000
2	3,333	2,886.751	41,667	5,773.502	23,333	2,886.751
3	6,667	2,886.751	48,333	10,408.33	16,666	2,886.751
4	11,667	5,773.502	151,667	40,414.520	138,333	18,930.000
5	75,000	10,000.000	160,000	10,000.000	148,333	34,034.000
6	28,333	2,886.751	210,000	65,000.000	176,667	32,532.035
7	50,000	10,000.000	156,667	52,041.650	208,333	28,431.200
8	40,000	15,000.000	141,667	27,537.850	128,333	20,207.260
9	21,667	10,408.000	143,333	12,583.060	241,667	32,532.035

## References

[1] Choi H.Y., Park K.S., Park S.J., Paek U.-C., Lee B.H.E., Choi S. (2008). Miniature fiber-optic high temperature sensor based on a hybrid structured Fabry–Perot interferometer. Opt. Lett..

[2] De la Rosa E., Zenteno L.A., Starodumov A.N., Monzon D. (1997). All-fiber absolute temperature sensor using an unbalanced high-birefringence Sagnac loop. Opt. Lett..

[3] Yasin M., Harun S.W., Abdul-Rashid H.A., Kusminarto Karyano, Ahmad H. (2008). The performance of a fibre optic displacement sensor for different types of probes and targets. Las. Phys. Lett..

[4] Lim K.S., Moghaddam M.R.A., Harun S.W., Ahmad H. (2010). Tunable-Spacing Multiwavelength Yb-doped Fibre Laser (YDFL) based on temperature sensitive loop mirror. Laser Eng..

[5] Lee B. (2003). Review of the present status of optical fibre sensors. Opt. Fibre Technol..

[6] Ahmad H., Chong W.Y., Thambiratnam K., Zulklifi M.Z., Poopalan P., Thant M.M.M., Harun S.W. (2009). High sensitivity fiber Bragg grating pressure sensor using thin metal diaphragm. IEEE Sens. J..

[7] Brogan K.L., Walt D.R. (2005). Optical fiber-based sensors: Application to chemical biology. Curr. Opin. Chem. Biol..

[8] El-Sherif M., Bansal L., Yuan J. (2007). Fiber optic sensors for detection of toxic and biological threats. Sensors.

[9] Grattan K.T.V., Sun T. (2000). Fiber optic sensor technology: An overview. Sens. Actuators A Phys..

[10] Kersey A.D. (1996). A review of recent developments in fibre optic sensor technologies. Opt. Fibre Technol..

[11] Marquet P., Rappaz B., Magistretti P.J., Cuche E., Emery Y., Colomb T., Depeursinge C. (2005). Digital holographic microscopy: A noninvasive contrast imaging technique allowing quantitative visualization of living cells with subwavelength axial accuracy. Opt. Lett..

[12] Anand A., Chhaniwal V.K., Javidi B. (2011). Imaging embryonic stem cell dynamics using quantitative 3-D digital holographic microscopy. IEEE Photon. J..

[13] Kuo Y.C., Ho J.H., Yen T.J., Chen H.F., Lee O.K. (2011). Development of a surface plasmon resonance biosensor for real-time detection of osteogenic differentiation in live mesenchymal stem cells. PLoS One.

[14] Wood M.F., Ghosh N., Wallenburg M.A., Li S.H., Weisel R.D., Wilson B.C., Li R.K., Vitkin I.A. (2010). Polarization birefringence measurements for characterizing the myocardium, including healthy, infarcted, and stem-cell-regenerated tissues. J. Biomed. Opt..

[15] Ramsden J.J., Horvath R. (2009). Optical biosensors for cell adhesion. J. Recept. Signal Transduct..

[16] Aref A., Horvath R., McColl J., Ramsden J.J. (2009). Optical monitoring of stem cell-substratum interactions. J. Biomed. Opt..

[17] Robertson J.A. (2010). Embryo stem cell research: ten years of controversy. J. Law Med. Ethics.

[18] Pittenger M.F., Mackay A.M., Beck S.C., Jaiswal R.K., Douglas R., Mosca J.D., Moorman M.A., Simonetti D.W., Craig S., Marshak D.R. (1999). Multilineage potential of adult human mesenchymal stem cells. Science.

[19] Le Blanc K. (2003). Immunomodulatory effects of fetal and adult mesenchymal stem cells. Cytotherapy.

[20] Batsali A.K., Kastrinaki M.C., Papadaki H.A., Pontikoglou C. (2013). Mesenchymal Stem Cells derived from Wharton's Jelly of the Umbilical Cord: Biological properties and emerging clinical applications. Curr. Stem Cell Res. Ther..

[21] López Y., Lutjemeier B., Seshareddy K., Trevino E.M., Hageman K.S., Musch T.I., Borgarelli M., Weiss M.L. (2013). Wharton's jelly or bone marrow mesenchymal stromal cells improve cardiac function following myocardial infarction for more than 32 weeks in a rat model: A preliminary report. Curr. Stem Cell Res. Ther..

[22] Gronthos S., Brahim J., Li W., Fisher L.W., Cherman N., Boyde A., DenBesten P., Gehron Robey P., Shi S. (2002). Stem cell properties of human dental pulp stem cells. J. Dent. Res..

[23] Gronthos S., Mankani M., Brahim J., Robey P.G., Shi S. (2000). Postnatal human dental pulp stem cells (DPSCs) *in vitro* and *in vivo*. Proc. Natl. Acad. Sci. USA.

[24] Seo B.-M., Miura M., Gronthos S., Bartold P.M., Batouli S., Brahim J., Young M., Robey P.G., Wang C.Y., Shi S. (2004). Investigation of multipotent postnatal stem cells from human periodontal ligament. The Lancet.

[25] Sonoyama W., Liu Y., Fang D., Yamaza T., Seo B.-M., Zhang C., Liu H., Gronthos S., Wang C.-Y., Shi S., Wang S. (2006). Mesenchymal stem cell-mediated functional tooth regeneration in swine. PLoS One.

[26] Govindasamy V., Abdullah A.N., Ronald V.S., Musa S., Ab. Aziz Z.A.C., Zain R., Totey S., Bhonde R.R., Abu Kasim N.H. (2010). Inherent differential propensity of dental pulp stem cells derived from human deciduous and permanent teeth. J. Endod..

[27] Friedenstein A.J., Gorskaja J.F., Kulagina N.N. (1976). Fibroblast precursors in normal and irradiated mouse hematopoietic organs. Exp. Hematol..

[28] Collas P. (2010). Programming differentiation potential in mesenchymal stem cells. Epigenetics.

[29] Nekanti U., Rao V.B., Bahirvani A.G., Jan M., Totey S., Ta M. (2010). Long-term expansion and pluripotent marker array analysis of Wharton's Jelly-derived mesenchymal stem cells. Stem Cells Dev..

[30] Xu W., Huang X.G., Pan J.S. (2013). Simple fiber-optic refractive index sensor based on fresnel reflection and optical switch. IEEE Sens. J..

[31] Hsiao S.T., Asgari A., Lokmic Z., Sinclair R., Dusting G.J., Lim S.Y., Dilley R.J. (2012). Comparative analysis of paracrine factors expression in human adult mesenchymal stem cells derived from bone marrow, adipose and dermal tissue. Stem Cells Dev..

[32] Yeun C.T., Rizon M., Shazri M. (2009). Real-tme detection of face and iris. WSEAS Trans. Signal. Process..

[33] Chuah Z-M., Paramesran R., Thambiratnam K., Poh S.C. (2010). A two-level partial least squares system for non-invasive blood glucose concentration prediction. Chemom. Intell. Lab. Syst..

